# High-fat diet promotes tumor growth in the patient-derived orthotopic xenograft (PDOX) mouse model of ER positive endometrial cancer

**DOI:** 10.1038/s41598-023-43797-1

**Published:** 2023-10-02

**Authors:** Ke Shen, Dandan Shen, Dongdong Jin, Yichao Zheng, Yuanhang Zhu, Xinyue Zhao, Zhenan Zhang, Nannan Wang, Huanhuan Chen, Li Yang

**Affiliations:** 1 Department of Obstetrics and Gynecology, The Third Affiliated Hospital of Zhengzhou University, Zhengzhou, China; 2 Key Laboratory of Advanced Drug Preparation Technologies, Ministry of Education of China, Key Laboratory of Henan Province for Drug Quality and Evaluation, Institute of Drug Discovery and Development, School of Pharmaceutical Sciences, Zhengzhou University, 100 Kexue Avenue, Zhengzhou, 450052 Henan China; 3 State Key Laboratory of Esophageal Cancer Prevention & Treatment; Academy of Medical Sciences, Zhengzhou University, 100 Kexue Avenue, Zhengzhou, Henan China; 4 Zhengzhou Key Laboratory of Endometrial Disease Prevention and Treatment, Zhengzhou, China

**Keywords:** Cancer models, Endometrial cancer, Pathogenesis, Cancer genomics

## Abstract

Endometrial cancer, one of the common gynecological malignancies, is affected by several influencing factors. This study established a unique patient-derived orthotopic xenograft (PDOX) nude mouse model for the study of influencing factors in ER positive endometrial cancer. The aim of this study was to demonstrate that a high-fat diet can affect the growth of ER positive endometrial cancer PDOX model tumors. The tumor tissues were expanded by subcutaneous transplantation in nude mice, and then the subcutaneous tumor tissues were orthotopically implanted into the nude mouse uterus to establish the PDOX model. After modeling, they were divided into high-fat diet group and normal diet group for 8 weeks of feeding, which showed that high-fat diet significantly promoted tumor growth (P < 0.001) and increased the protein expression level of ERα in tumor tissues. This study demonstrates that PDOX models of endometrial cancer can embody the role of dietary influences on tumor growth and that this model has the potential for preclinical studies of cancer promoting factors.

## Introduction

Endometrial cancer (EC), a malignant tumor of the endometrial epithelium, is one of the most common gynecological malignancies^1^. With increasing global incidence and disease-related mortality, endometrial cancer has a global incidence of about 417 thousand in 2020 and becomes the sixth most common female cancer^2^. As the most common gynecological cancer in high income countries, its incidence is rising globally^3^. The pathogenesis and influencing factors of endometrial cancer are still topics that require continued intensive research.

The relationship between the intake of a high-fat diet and the risk of endometrial cancer is so far inconclusive. Studies have shown that obesity is a major risk factor for endometrial cancer^4,5^, International Agency for Research on Cancer also states that higher fat mass is a cause of endometrial cancer^6^. It is generally accepted that adipocytes can convert androgens into estrogens to increase estrogen production and thereby stimulate endometrial proliferation and cancer development^7^. It has also been shown that higher fat intake is associated with increased plasma estradiol, insulin secretion and IGF levels as well as inflammatory markers, suggesting that dietary fat intake may promote endometrial carcinogenesis by altering estrogen, insulin and IGF, and the inflammatory system^8–10^. But it has also been shown that total intake of animal and plant fats is not associated with endometrial cancer^11^. Estrogen signaling pathway and estrogen receptors play important roles in endometrial carcinogenesis^12,13^. Estrogen receptor α, (ERα) is a steroid hormone receptor expressed in the vast majority (> 85%) of type I endometrial carcinomas^14^. Estrogen receptor has been found to play a key role in breast cancer carcinogenesis as a coregulator of gene expression^15,16^. It has also been reported in endometrial cancer that ERα is an important factor for endometrial cancer cell growth^17^. We speculated that high-fat diet might directly affect the expression level of ERα in ER positive endometrial cancer tumor tissues and thus influence the development of endometrial cancer.

Patient-derived xenograft (PDX) models, in which fresh cancer patient tissue samples are cut directly into small pieces or broken down into cell suspensions and surgically implanted or inoculated into immune-compromised mice^18^, enable several generations of sequential amplification to maintain a high degree of histopathology, genetic heterogeneity and growth characteristics of the primary tumor, preserving the genetic fidelity of the tumor^19,20^. In contrast to subcutaneous transplantation, the patient-derived orthotopic xenograft (PDOX) model is worth trying because it selects the same organ for transplantation as the primary tumor, has the same growth environment as the primary tumor, and is able to simulate a variety of biological behaviors including growth, infiltration, and metastasis, and are more conducive to the occurrence of tumor metastasis due to the abundant blood supply at the transplantation site^21–23^.The use of tumor fragments rather than cell suspensions preserves the structural integrity of the tissue and retains cell–cell interactions and therefore better mimics the tumor microenvironment^24–26^. Therefore, the use of this model in preclinical studies of cancer risk factors will more realistically simulate the development of tumors in vivo, resulting in more reliable findings that are more in line with clinical realities.

In this study, we established a PDOX model of endometrial cancer by directly implanting fragments of ER positive endometrial cancer into the uterus of nude mice after subcutaneous amplification, and for the first time, chose to explore the effect of a high-fat diet on tumor growth in an endometrial cancer PDOX animal model. The PDOX model was able to recapitulate the effects of an 8-week high-fat diet on tumor growth, with the high-fat diet promoting tumor growth and increasing ERα protein expression levels in tumor tissues. This demonstrates that studies on cancer influencing factors can be performed with this endometrial cancer PDOX model that more closely mimics the in vivo real environment.

## Materials and methods

### Experimental animals and tissue samples

Thirty healthy female 4–5 weeks BALB/C nude mice, weighing 16–18 g, were provided by SiPeiFu (Beijing) Biotechnology Co. The nude mice were housed in the SPF class animal room of Zhengzhou University Experimental Animal Center at an environmental temperature of 24–26 °C, relative humidity of 50–60%, 12 h of light and 12 h of darkness. All mice were given free access to water and food, and were fed with autoclaved water and chow. Animal experiments were approved by the Ethics Committee of Zhengzhou University Laboratory Animal Center (202109140101).

Six patients with ER-positive endometrial cancer underwent hysterectomy at the Third Affiliated Hospital of Zhengzhou University. Basic patient information is shown in the Table 1. Patients provided written informed consent and the Ethics Committee of the Third Affiliated Hospital of Zhengzhou University approved this experiment (2022-216-01). Experiments in the present study were performed per the Declaration of Helsinki guidelines and in agreement with national regulations for the experimental use of human material. All methods were performed in accordance with the relevant guidelines and regulations. The study is reported in accordance with ARRIVE guidelines (https://arriveguidelines.org).

**Table 1:** Basic patient information for tumor transplantation in endometrial cancer animal models.

Number	Clinical diagnosis	Age	Postoperative pathology	Degree of differentiation	Immunohistochemistry
1	Endometrial cancer stage Ib	52	Endometrioid carcinoma with infiltration depth > 1/2 full thickness of uterine wall	Moderately differentiated	ER (+)
2	Endometrial cancer stage Ia	69	Endometrioid carcinoma, infiltrating the superficial myometrium of the uterine wall (< 1/2 of the uterine wall)	Moderately-poorly differentiated	ER (+)
3	Endometrial cancer stage Ib	45	Endometrioid carcinoma with infiltration depth > 1/2 full thickness of uterine wall	Moderately-poorly differentiated	ER (+)
4	Endometrial cancer stage Ia	31	Endometrioid carcinoma, infiltrating the superficial myometrium of the uterine wall (< 1/2 of the uterine wall)	Poorly differentiated	ER (+)
5	Endometrial cancer stage Ia	58	Endometrioid carcinoma, infiltrating the superficial myometrium of the uterine wall (< 1/2 of the uterine wall)	Moderately differentiated	ER (+)
6	Endometrial cancer stage Ia	63	Endometrioid carcinoma, infiltrating the superficial myometrium of the uterine wall (< 1/2 of the uterine wall)	Well differentiated	ER (+)

### Construction of PDOX model of EC in nude mice

#### Construction of PDX model

**Figure 1:**
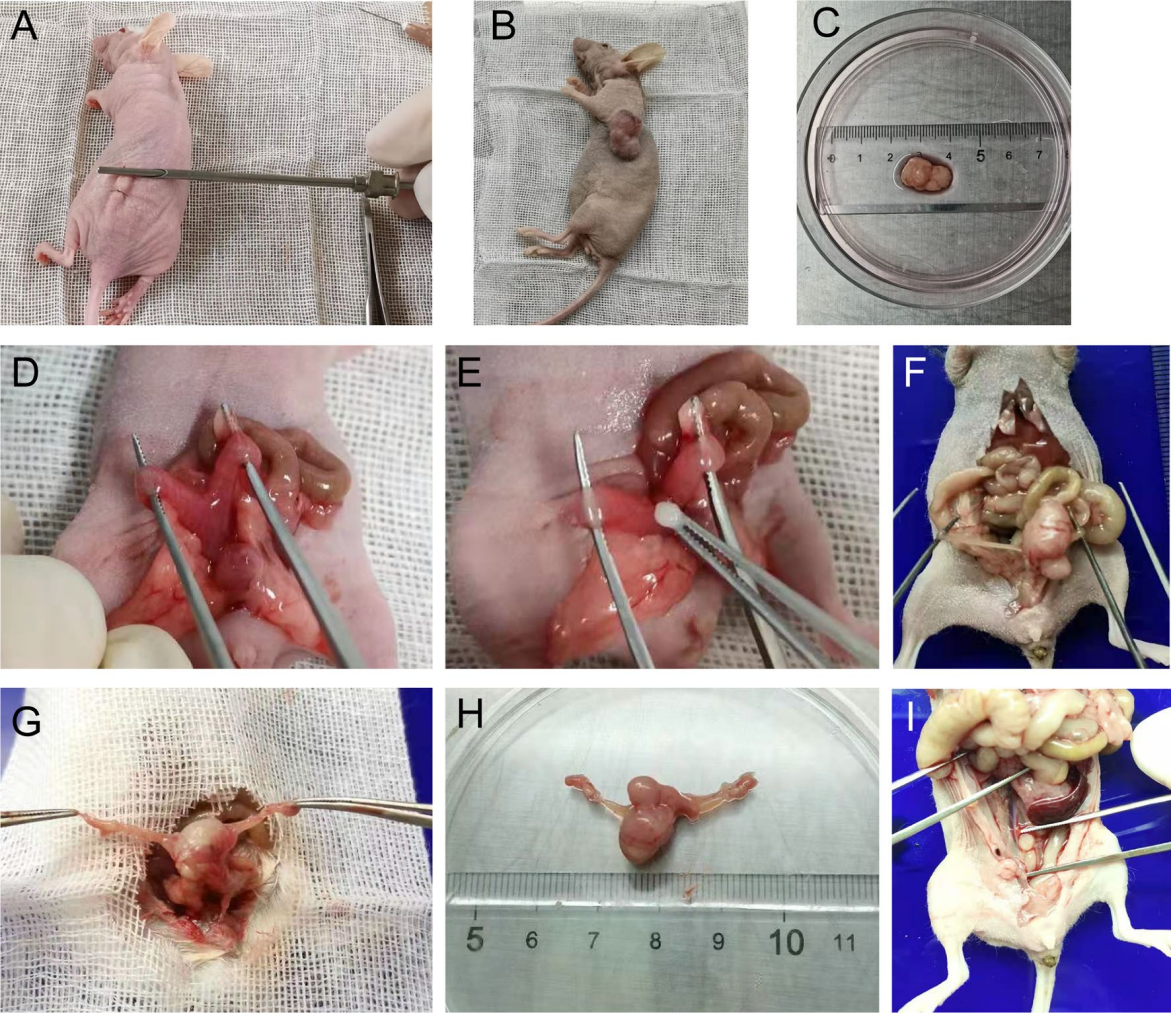
Construction of PDOX model of EC. (**A**) Patient-derived histologically intact EC tissue was implanted subcutaneously using a sleeve needle. (**B**) Successful subcutaneous tumor transplantation in nude mice. (**C**) Isolated subcutaneous tumors were placed in sterile saline. (**D**) The Y-shaped uterus of the nude mouse was fully exposed. (**E**) A tissue block of approximately 1 mm^3^ is implanted at the Y-shaped uterine bifurcation. (**F**,**G**) Macroscopic view of tumor formation after 6 weeks of orthotopic xenograft. (**H**) Tumors and new blood vessel formation can be seen. (**I**) Suspected metastases were found in the pelvis.

Fresh tissues were rinsed in sterile saline at 4 °C and then immediately transferred to a biosafety cabinet for manipulation. After removing the connective tissue and necrotic tissue, the tumor tissue was cut into 1 mm^3^ pieces, and one piece of tumor tissue was selected and sucked into the sleeve needle. After intraperitoneal injection of 1% pentobarbital sodium (Sigma, America) for anesthesia, the mice were placed in the prone position to clean the skin of the axilla or hind limb and disinfected. Use ophthalmic scissors to cut out a small 2 mm needle entry opening, push the sleeve needle (Kebang Biotechnology, Zhengzhou, China) from the incision to the inoculation site, insert the needle core to push the tissue block out of the sleeve and place it under the skin, fix the needle core and pull out the sleeve, and finally slowly pull out the needle core to complete inoculation (Fig. 1A). One stitch of 5-0 absorbable suture (Kebang Biotechnology, Zhengzhou, China) was used to close the incision site and penicillin drops were applied to the incision site to prevent infection. Postoperatively, weight and subcutaneous tumor growth were recorded weekly.

#### Construction of PDOX model

After the tumors grew to more than 1 cm in length and diameter in the subcutaneously transplanted mice described above, the mice were sacrificed by cervical dislocation and the tumors were removed and cut into 1 mm^3^ pieces in the same way (Fig. 1B,C). Use ophthalmic scissors to make a longitudinal incision of about 1 cm from the midline of the abdomen, turn up the intestinal tube that blocks the view and expose the uterus (Fig. 1D). A small incision of approximately 2 mm was made in the entire uterine wall at the Y-shaped bifurcation of the uterus, and one piece of tumor tissue was implanted in the uterine cavity at the fundus (Fig. 1E). The uterus was closed with 1 stitch of 5-0 absorbable suture, and the abdomen was closed layer by layer, and penicillin was placed in the incision to prevent infection. After the operation, the body weight and the degree of abdominal distension were observed weekly, and the mice were executed when they were in a dying state and systematically dissected.

### Group feeding and tumor processing

The modeled nude mice were randomly divided into a HFD group and a ND group, with 10 mice in each group. The mice in the HFD group were fed with a high fat feed (D12492, 5.24 kcal/g, 60% fat energy supply) ^27,28^and the mice in the ND group were fed with a normal mouse growth maintenance diet (3.85 kcal/g, 12% fat energy supply) for 8 weeks. High fat feed was provided by SiPeiFu (Beijing) Biotechnology Co., Ltd. Twenty percent of the total energy produced by the feed is provided by protein, 20% by carbohydrates, and 60% by fat. 90% of the energy supplied by fat comes from lard, which is a typical representative of saturated fatty acids. The major fatty acids are palmitic acid (a kind of high saturated fatty acid), oleic acid and linoleic acid. The other 10% of energy comes from soybean oil, which mainly provides unsaturated fatty acids. All mice were weighed weekly, and the mice were executed after 8 weeks. The body weight and body length were measured and recorded for both groups, and Lee's index (Lee's index = weight (g)^(1/3)^ × 1000/body length (cm)) was calculated for both groups. The tumor morphology of EC was observed, and the tumor volume (V = π/6 × L × W^2^(mm^3^)) was calculated after measurement with vernier calipers. Tumor specimens were fixed in formalin and embedded in paraffin or directly stored at − 80°℃.

### Tumor histopathological examination and immunohistochemistry

The model mice whose tumor length reached 1 cm were euthanized by cervical dislocation method, and the tumor tissues were stripped for photo recording. Appropriate amounts of tumor tissue were fixed in a 10% neutral formaldehyde solution for at least 24 h, dehydrated through graded alcohols and cleared through xylene, embedded in soaked wax, and sectioned after the tissue blocks had become rigid (4 μm), attach to glass slides and dry. Deparaffinization using xylene before staining and final staining through graded alcohols to distilled water. For staining, the slides were counterstained in hematoxylin for 2 min, acid water and ammonia water for 2 min, alcohol eosin for 2 min, and dehydrated. The HE stained tissue sections were observed under a light microscope and scanned for the staining results.

Immunohistochemistry to determine whether ER was positive, tissue sections were deparaffinized in xylene, rehydrated in graded ethanol, and soaked in distilled water. After heat mediated citrate antigen retrieval, tissue sections were incubated with primary antibody for Era (GB111843, 1:1000; servicebio) overnight at 4 °C and with horseradish peroxidase conjugated antibody for 60 min at room temperature. Color was developed by incubation with diaminobenzidine. Finally, these treated tissue sections were observed under a microscope and then counterstained with hematoxylin. Negative versus positive results were independently adjudicated by two pathologists.

### Scanning PDOX models using small animal ultrasound

After 6 weeks of transplantation, most of the model mice significantly touched the formation of orthotopic transplanted tumors in the abdomen. The mice were anesthetized with isoflurane gas (a mixture of 2% isoflurane and O_2_ gas), and then placed in a hood connected to a gas anesthetic for fixation. The length, width, depth, and maximum cross-sectional area of each nude mouse endometrial cancer tumor were measured by ultrasound scanning using a small animal Vevo^®^ 2100 ultrasound imaging platform (Japan) purchased by Zhengzhou University Laboratory Animal Center. Mice rapidly returned to wakefulness after the measurement ended.

### RNA-sequencing and data analysis

UID RNA-seq experiment and high through-put sequencing and data analysis were conducted by Seqhealth Technology Co., LTD (Wuhan, China). After a primary test for RNA quality and integrity, the sequencing samples were finally sequenced on Novaseq 6000 sequencer (Illumina) with PE150 model. The raw sequencing data were mapped to the human genome using STRA 2.5 software. Reads mapped to the exon regions of each gene were counted by featureCounts, and then reads per kilobase of transcript per million reads mapped was calculated^29,30^. Differentially expressed (p-value cutoff of 0.05; the fold-change cutoff of 2) genes between groups were identified using the edgeR package (version 3.12.1)^31,32^. Gene ontology (GO) analysis and Kyoto encyclopedia of genes and genomes (KEGG) enrichment analysis for differentially expressed genes were both implemented by KOBAS software (version: 2.1.1) with a P-value cutoff of 0.05 to judge statistically significant enrichment^33^.

### Western blot analysis

Tissue proteins were extracted using RIPA lysis buffer solution (Servicebio, Wuhan, China) containing PMSF (Servicebio, Wuhan, China) at a ratio of 100:1. After incubation on ice for 30 min, the tissue homogenate was centrifuged at 12,000*g* for 10 min and the protein-containing supernatant was collected. Protein extracts were separated by sodium dodecyl sulfate–polyacrylamide gel electrophoresis (SDS-PAGE), and transferred to PVDF membranes (Servicebio, Wuhan, China). Then, after blocking the membranes with skim milk (5%), the membrane blots were incubated at 4 °C overnight with primary antibodies: ERA (GB111843, 1:1,000; Servicebio), GAPDH (GB15003, 1:3000; Servicebio). GAPDH was used as an endogenous control. Subsequently, the membranes were incubated with the HRP-conjugated secondary antibody for 1 h at room temperature. Immunoreacted protein bands were detected with the NcmECL Ultra detection system (New Cell & Molecular Biotech. Co., Ltd., Suzhou, China) on a Bio-Rad Chemi-Doc XRS system (Bio-Rad, Hercules, CA, USA) with automatic exposure control.

### Statistical analysis

Data were analyzed using the SPSS software (IBM Corporation, Armonk, NY, USA) and Graphpad Prism 8.0 (GraphPad Software Inc., San Diego, CA, USA). All the experimental data were collected from at least three repeated experiments, if not otherwise stated. The data are presented as the mean ± SD and the p-value < 0.05 was considered to indicate statistically significant difference.

## Results

### Successful endometrial cancer PDOX model construction in BALB/C nude mice

The endometrial cancer PDOX model was successfully constructed, and the mice were in good condition during the construction process, with normal feeding and water intake. There was less intraoperative bleeding, no need for hemostasis, and the visual field was clear. After 12 h post operation, the mice can be normal activity and diet, mental status is good. After 6–8 weeks, the tumor mass increased significantly, and on gross view, gray white tumor body tissue with a raised appearance was visible upon dissection of subcutaneous tumorigenesis, the tumor appeared nodular, outsourced an intact connective tissue envelope, and the tumor body had no obvious adhesion to surrounding tissues (Fig. 1B,C). Dissection for orthotopic tumorigenesis revealed an appearance of raised gray white body tumor tissue with proliferation of surrounding blood vessels and a nodular appearance of the tumor. Adhesion of the tumor to the periuterine tissue was found in 65% (13/20) of mice, and satellite metastases formation around the tumor body was observed in 40% (8/20) of mice (Fig. 1F–I).

### The model tumors maintained histopathological morphology and molecular features with the original tumors

**Figure 2:**
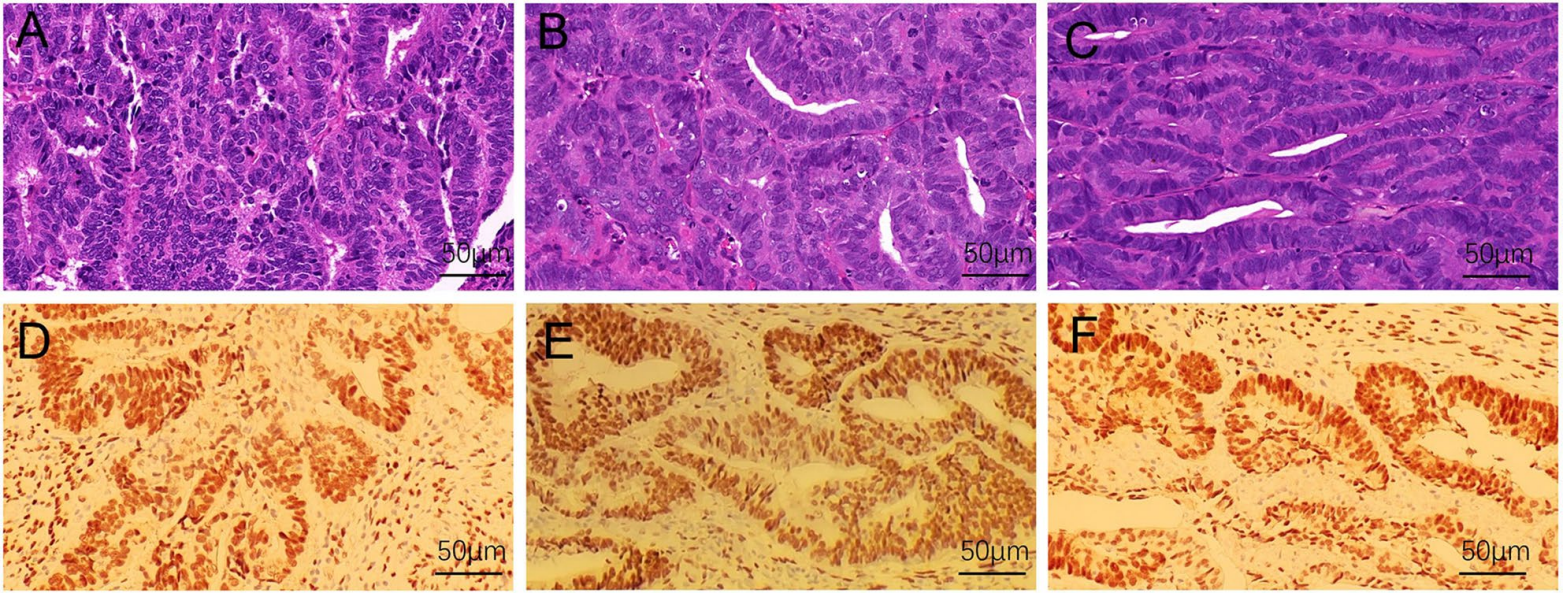
Histopathological morphology observation of tumors from endometrial cancer patients and transplanted tumors in nude mice. (**A**) HE staining of patient tumor tissue. (**B**) HE staining of subcutaneous tumorigenic tissues in PDX model. (**C**) HE staining of uterine tumorigenic tissues in PDOX model. (**D**) IHC showed ER expression in patient tumor tissue. (**E**) IHC showed ER expression in PDX model. (**F**) IHC showed ER expression in PDOX model.

After the EC PDX model and PDOX model were successfully formed, the tumor tissue of EC patients, the tumor tissue formed by subcutaneous transplantation in nude mice and the tumor tissue of orthotopic xenograft in nude mice were observed under light microscope respectively. HE staining of the three tissues showed glandular growth of tumor cells in close arrangement with poor polarity, high nuclear cytoplasmic ratio, and mild heterogeneity, and massive nuclear division, focal necrosis foci, nuclear pyknosis, nuclear fragmentation and karyolysis and capillary congestion were observed, overall inflammatory cell infiltration was not obvious (Fig. 2A–C). ER expression was demonstrated by immunohistochemistry in all three tissues (Fig. 2D–F). The results of HE staining and immunohistochemistry of both subcutaneous and orthotopic tumors in the PDOX model of endometrial cancer remained highly consistent with the original tumor of the patient, indicating that we successfully constructed the PDOX model of endometrial cancer in BALB/C nude mice, which can better maintain the tissue structure and molecular characteristics of patient-derived tumor tissues.

### Endometrial cancer PDOX models have higher rates of tumorigenesis than subcutaneous models

**Figure 3:**
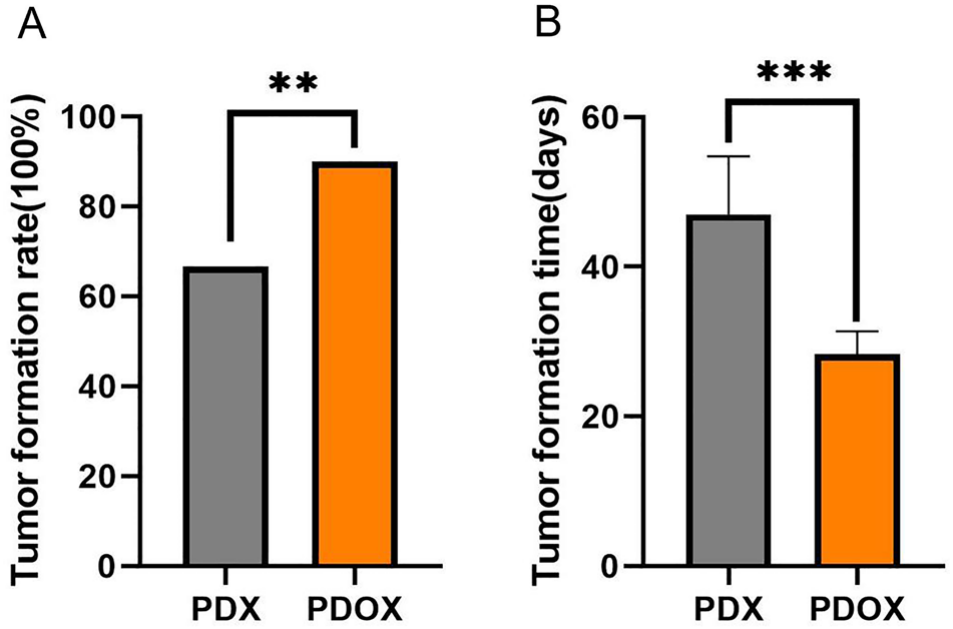
Tumorigenesis rate and time to tumorigenesis in a subcutaneous PDX model of endometrial cancer and an orthotopic PDOX model. (**A**) Tumorigenesis rates of PDX models versus PDOX models. (**B**) Tumorigenic time of PDX models versus PDOX models. PDX (n = 12), PDOX (n = 20). Data are shown as the mean ± SD. ***P < 0.001, **P < 0.01.

The tumorigenic rate and tumorigenic cycle of PDOX models are two important indicators that constrain the application of the models. In this experiment, 6 patient-derived endometrial cancer tissues were transplanted subcutaneously into the left armpit and hind limb of 6 female BALB/C nude mice, and a total of 12 sites were transplanted, with 8 sites of tumorigenesis, and the overall success rate of PDX modeling was 66.7% (8/12). Uterine orthotopic modeling was performed on 20 syngeneic nude mice using tumor tissues from subcutaneous tumorigenic sites, of which one mouse died during modeling due to surgical error, one mouse was not tumorigenic, and the endometrial cancer bodies in the remaining mice grew well and obviously enlarged, and the overall success rate of PDOX modeling was 90% (18/20) (Fig. 3A). Small nodules could be observed subcutaneously in PDX models after 4 weeks of transplantation, and obvious growth of tumor tissue was observed at 6 weeks, whereas hard nodules were palpable in the abdomen as soon as 2–3 weeks for PDOX models, and obvious growth of transplanted tumors was observed from the abdomen as soon as 4 weeks (Fig. 3B). The comparison of the subcutaneous PDX model and the orthotopic PDOX model of endometrial cancer is shown in Table 2.

**Table 2:** Endometrial cancer PDX models compared with PDOX models.

Type	PDX	PDOX
Surgical procedures	Simple	Complex
Tumor detection	Vernier caliper measurements	Ultrasonography
Tumorigenesis rate	Low	High
Time to tumorigenesis	Longer	Shorter
Histopathology	High similarity	High similarity

### High fat diet promotes body weight and tumor growth in PDOX model of endometrial cancer

**Figure 4:**
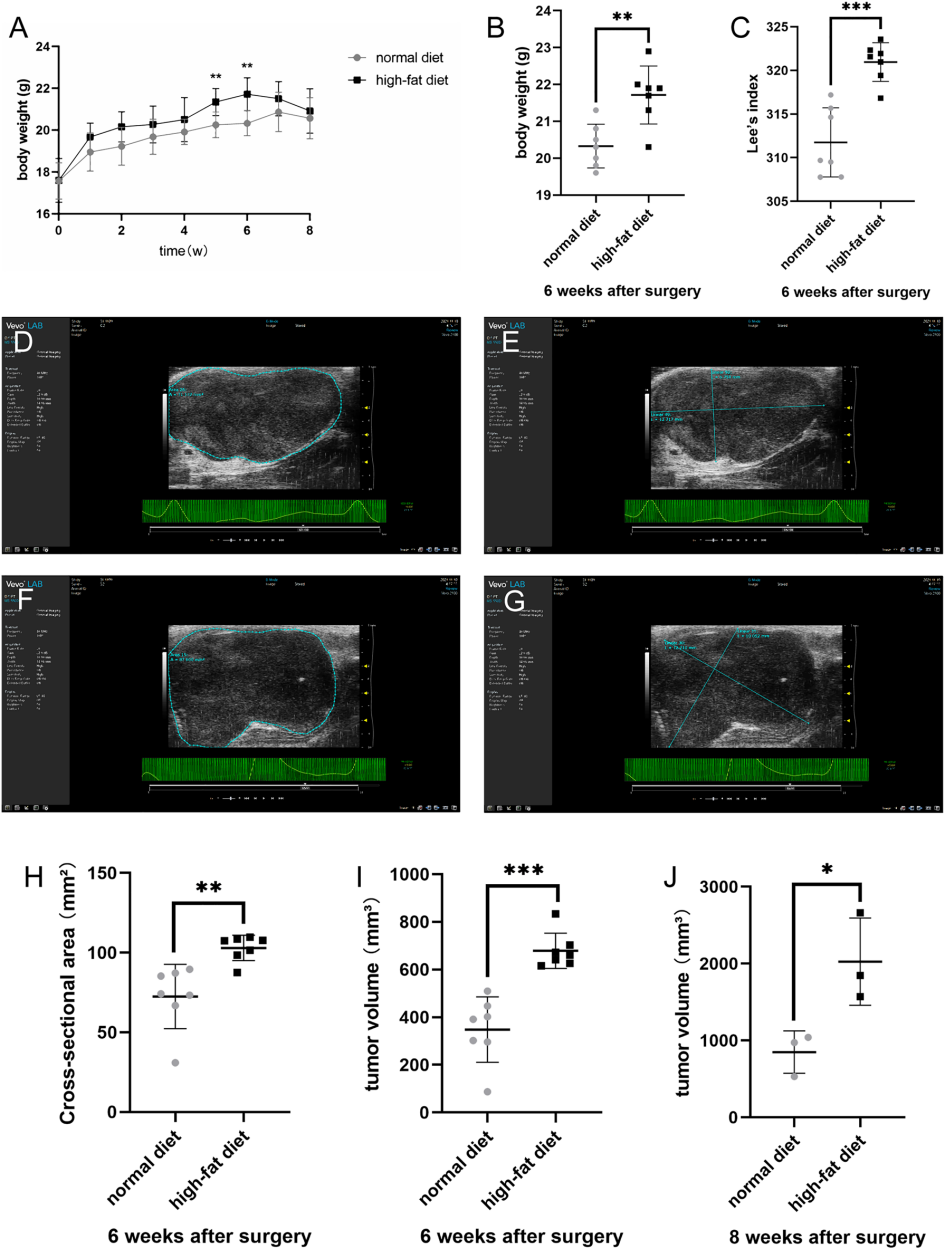
Body weight, Lee’s index, Cross-sectional area and tumor volume were statistically significant after modeling. (**A**) Weight change curve of nude mice. (**B**) Body weight of nude mice 6 weeks after transplant surgery. (**C**) Lee’s index of nude mice 6 weeks after transplant surgery. (**D**,**E**) Ultrasound images of tumors in nude mice of ND group 6 weeks after transplant surgery. (**F**,**G**) Ultrasound images of tumors in nude mice of HFD group 6 weeks after transplant surgery. (**H**) Tumor cross-sectional area of nude mice on ultrasound 6 weeks after transplant surgery. (**I**) Tumor volume of nude mice 6 weeks after transplant surgery. (**J**) Tumor volume of nude mice 8 weeks after transplant surgery. Data are shown as the mean ± SD. ***P < 0.001, **P < 0.01, *P < 0.05.

**Figure 5:**
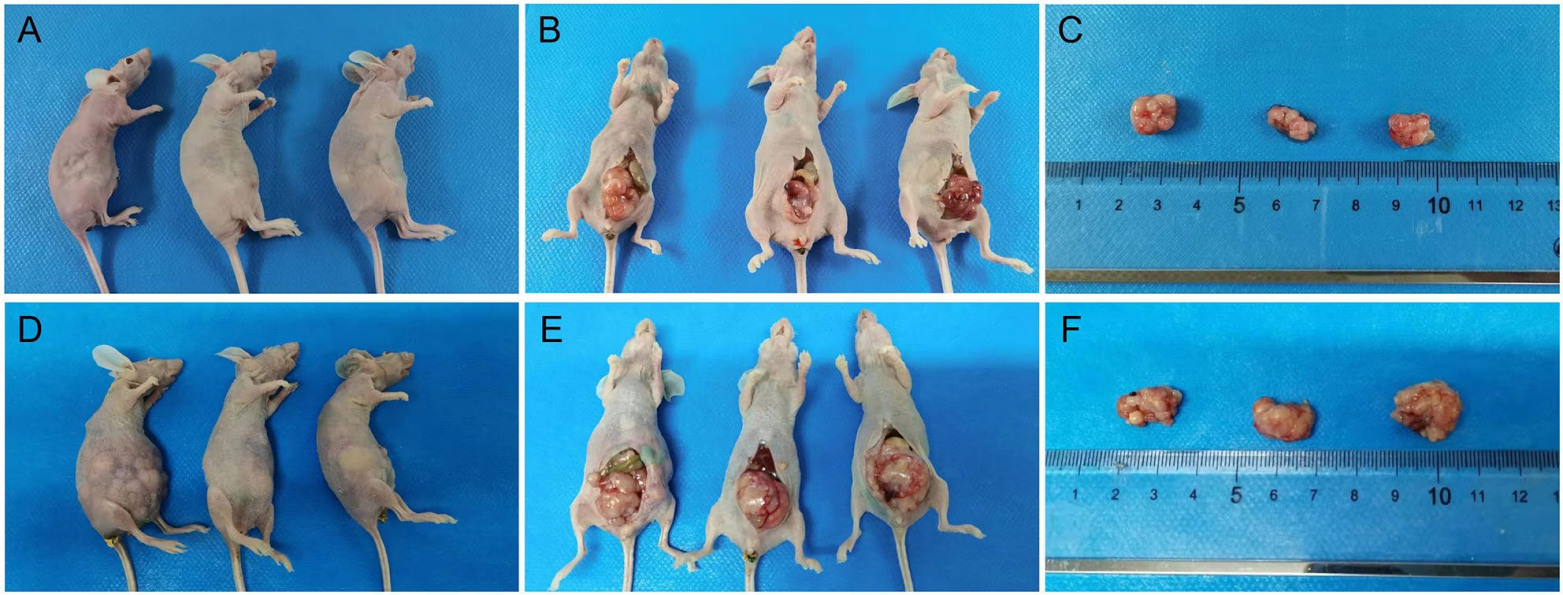
Nude mice after transplantation and group feeding for 8 weeks. (**A**–**C**) Nude mice, the uterus and tumor, tumor in vitro view of normal diet group. (**D**–**F**) Nude mice, the uterus and tumor, tumor in vitro view of high-fat diet group.

Regular measurements of body weight and length of the mice showed that there was no significant difference in the initial body weight of the mice before modeling, but the difference in body weight increased gradually from 5 weeks after modeling. The body weight of the high-fat diet group was larger than that of the normal diet group, with the most significant difference at 6 weeks after modeling (P < 0.01), as did the Lee's index (Table 3, Fig. 4A–C). Ultrasound probing of endometrial cancer lesions given at 6 weeks after modeling showed differences in both tumor cross-sectional area under ultrasound (Fig. 4D–H) and calculated tumor volume (Fig. 4I), as well as tumor volume measured 8 weeks after removing the tumor (Figs. 4J, 5). The cross-sectional area and volume of the tumor were larger in the high-fat diet group than in the normal diet group (P < 0.01).

**Table 3:** Body weight, Lee’s index, cross-sectional area and tumor volume were statistically significant after modeling.

	Body weight/g	Body weight 6 weeks after surgery/g	Lee’s index	Cross-sectional area/mm^3^	Tumor volume/mm^3^
Normal diet	17.57 ± 0.87	20.33 ± 0.59	311.75 ± 3.96	72.47 ± 20.17	348.01 ± 137.55
High-fat diet	17.60 ± 1.05	21.71 ± 0.79	320.95 ± 2.22	103.00 ± 7.93	678.88 ± 74.36
*t*	0.055	3.715	5.361	3.727	5.599
P	0.957	0.003	0.0002	0.003	< 0.0001

### Transcriptome sequencing of tumor tissues from high-fat diet group compared with normal diet group

**Figure 6:**
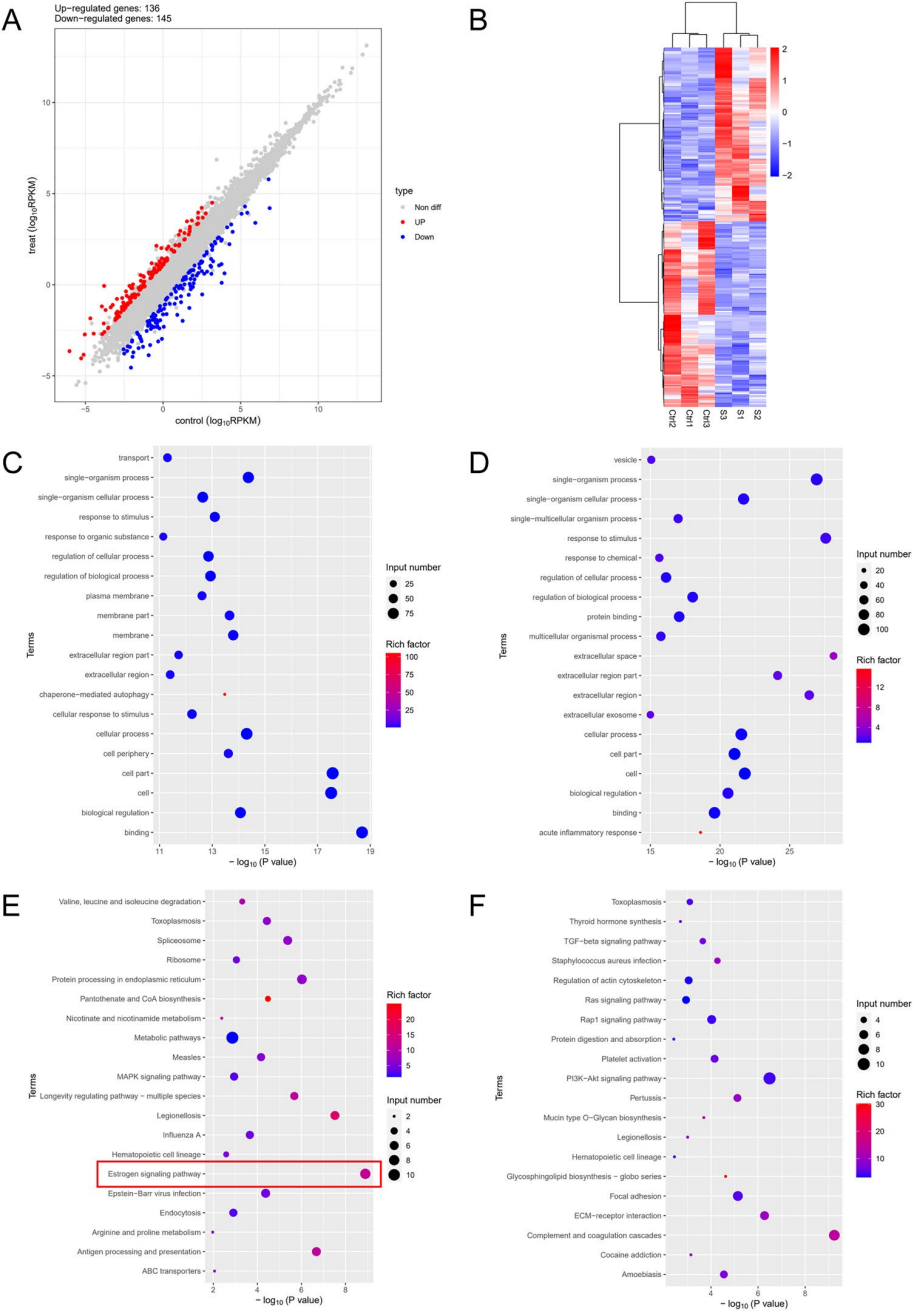
Differentially expressed genes between HFD and ND. (**A**) Scatter plot of differentially expressed genes between comparison groups. Compared to the ND group, blue dots represent differentially downregulated genes in HFD group, and red dots represent differentially upregulated genes in HFD group. (**B**) Overall hierarchical clustering diagram of all differentially expressed genes in comparison groups, clustered by reads per kilobase of transcript per million reads mapped value. Red represents high-expressed genes, and blue represents low-expressed genes. Ctrl represents ND group, and s represents HFD group. (STAR software (version 2.5.3a), edgeR package (version 3.12.1)). (**C**) GO enrichment map of upregulated genes. (**D**) GO enrichment map of downregulated genes. (**E**) KEGG enrichment map of upregulated genes. (**F**) KEGG enrichment map of downregulated genes. Clear images attached to supplementary information. Figure source: the KEGG software from the Kanehisa laboratory^46–48^.

RNA sequencing analysis was performed on tumor tissues obtained 8 weeks after modeling from high-fat diet group and normal diet group of mice. A total of 281 differentially expressed genes were screened under the conditions of P < 0.05 and |log2FC| > 1, of which 136 were up-regulated genes and 145 were down regulated genes (Fig. 6A). A clear distinction is seen in the Heatmap, with blue representing downregulated genes and red representing upregulated genes (Fig. 6B). Among the more significantly upregulated genes in the high-fat group were the ITGA4 gene, which plays a role in cell motility and migration^34,35^, the FSCN1 gene, which plays a role in metastasis of a variety of cancers^36–38^, the TWIST1 gene, which promotes tumor cell invasion and metastatic recurrence^39–41^, and the IGFBP2 gene, which promotes the growth of several tumors and predicts prognosis^42–44^, among others. The high expression of the above cancer promoting genes suggests that high-fat diet not only increases fat content to promote endometrial cancer tissue growth, but also plays a role in the transcription of gene molecules that promote tumor growth by upregulating the transcription levels of a battery of cancer promoting genes.

To further explore in which specific functions the differentially expressed genes cluster, we performed GO and KEGG analyses on these 281 genes. GO analysis results showed that the up-regulated genes were mainly involved in biological processes such as binding, biological regulation, chaperone mediated autophagy, and so on (Fig. 6C). The downregulated genes were mainly involved in extracellular space, response to stimulus, acute inflammatory response, and so on (Fig. 6D). KEGG analysis results showed that the main enrichment of upregulated genes was in the Estrogen signaling pathway (FKBP5, KCNJ5, HSPA8P5, HSPA8P8, HSPA8P9, HSP90AA6P), Antigen processing and presentation, Pantothenate and CoA biosynthesis, MAPK signaling pathway, Starch and sucrose metabolism, Insulin secretion, and so on (Fig. 6E). While the downregulated genes were mainly enriched in Complement and coagulation cascades, PI3K-Akt signaling pathway, Glycosphingolipid biosynthesis, Rap1 signaling pathway, and so on (Fig. 6F). Considering the role of estrogen signaling in endometrial cancer development^12,13,45^, after 8 weeks of high-fat diet administration to the PDOX animal model of endometrial cancer, the upregulated genes were obviously clustered in the estrogen signaling pathway, indicating that the promotion of endometrial cancer growth by high-fat diet is related to the estrogen signaling pathway, and the sustained high-fat diet may promote the progression of endometrial cancer by regulating the gene expression of estrogen signaling pathway at the genetic molecular level.

### Expression of ER proteins in tumor tissues from high-fat diet group and normal diet group

**Figure 7:**
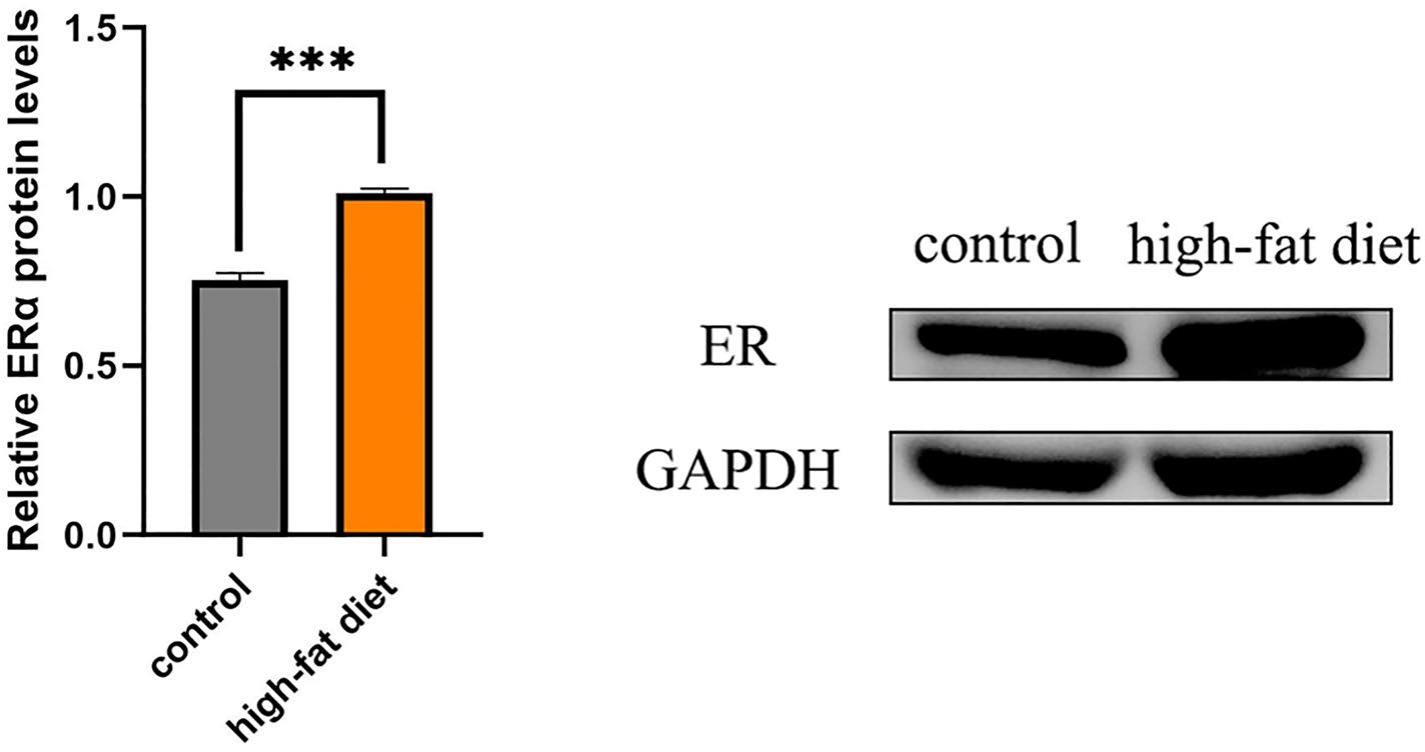
The amount of ERα protein expression was higher in the tumor tissues of the high-fat group than that in the normal diet group. Data are shown as the mean ± SD. ***P < 0.001.

Combined with the above sequencing analysis results about estrogen signaling pathway, we speculate that high-fat diet may also promote the expression of ERα in endometrial cancer tumor tissues in mice, thereby promoting the development of endometrial cancer. The expression of ERα proteins in the two groups of mouse endometrial cancer tumor tissues was determined using Western blot. The results showed that the expression of ERα proteins in the tumor tissues of the high-fat group was significantly higher than that in the normal diet group (P < 0.001) (Fig. 7). This indicates that high-fat diet indeed exerts its effect on endometrial cancer tumor tissues at both gene and protein expression levels. High fat diet upregulates ERα protein expression in endometrial cancer tumor tissues and promotes tumor growth of endometrial cancer.

## Discussion

The incidence rate of endometrial cancer is increasing^1,2^, and its pathogenesis and influencing factors are still topics that need further study. The development of PDOX model has been described in a variety of cancers^49,50^, including endometrial cancer. However, several steps of PDOX development are different among different researchers. Cabrera et al. first described an attempt to use an orthotopic PDX model of human endometrial carcinoma tumor tissue^51^. Haldorsen et al. developed an orthotopic PDX model by mechanically separating primary tumor into cell suspension^52^. Later, Pauli et al. described the development of PDX from patient-derived tumor-like organs (PDTO)^53^. The PDX model of 63 cases of endometrioid adenocarcinoma developed by European Network of Individual Treatment in Endometrial Cancer (ENITEC) members covers almost every stage and grade of the histological subtype of endometrioid adenocarcinoma^54^. Their sites include subcutaneous implantation and orthotopic implantation throughout a laparotomic incision. The engraftment rate of subcutaneous PDX models ranged from 60 to 80%, once the tumor grows, the engraftment rate increases to nearly 100% in subsequent generations. The PDX model takes about 3–5 months to get the first generation. In contrast, the implantation rate of the PDX model in situ is between 75 and 90%, and it also takes 2–5 months to form a palpable tumor.

In our modeling process, we observed the experimental results consistent with those of the ENITEC members. In terms of tumor formation rate, our PDX model has a tumor formation rate of 66.7% (8/12), and after the tumor grew and expanded subcutaneously, the tumorigenic rate of PDOX model reached 90% (18/20). In terms of tumorigenesis time, the first generation of effective subcutaneous tumorigenesis takes about 6–8 weeks. The PDOX model established after two subcutaneous expansions can develop palpable tumors in 3–4 weeks. The reason may be that in the fresh tissue from patients, tumor cells are scattered in the matrix tissue, resulting in a small number of tumor cells per unit area^25,55^. However, subcutaneously growing and subculturing in mice made tumor cells proliferate well, and obtained enough and high-quality tumor materials for orthotopic transplantation, which significantly increased the tumorigenesis rate and shortened the time required for tumorigenesis.

In this study, the PDOX animal model was used to investigate the pathogenesis and influencing factors of endometrial cancer for the first time. The endometrial cancer PDOX model we constructed, which well retains the characteristics of the patient's primary tumor in terms of histomorphology and molecular characteristics, is a good preclinical model suitable for scientific research. A high-fat diet for 6 weeks resulted in a significantly larger tumor volume, exemplifying the role of high-fat diet in promoting the development of endometrial cancer. And the ability of this model to demonstrate a clear response to a high-fat diet in the short term also exemplifies the potential of this model for studying factors influencing endometrial cancer growth and for in vivo studies of cancer promoting mechanisms.

In addition, we observed adhesions and metastases in orthotopically transplanted mice compared with subcutaneous transplants, which may be due to the more abundant blood supply to the orthotopically transplanted site, thus favoring the initiation of tumor metastasis^21–23^. This also demonstrates the power of PDOX models, perhaps through short-term model establishment, to identify the metastatic propensity of tumors and thus to make corresponding guidance for the clinical treatment of patients. Furthermore, probing drug response in PDOX models may have greater practical benefits for patients, and future studies will relate drug response in PDOX models to patients, perhaps to the development of drugs that target endometrial cancer induced by high-fat diets.

Illumina high-throughput sequencing revealed significant differences at the transcriptomic level between the high-fat diet group and the normal diet group, with high-fat diet upregulating genes including ITGA4, FSCN1, TWIST1 and IGFBP2 that play cancer promoting roles in various aspects of cancer cell growth, motile migration, invasion, metastatic recurrence as well as predicting prognosis. The results of KEGG analysis showed that the upregulated genes were obviously clustered in the estrogen signaling pathway (with minimal p-values and relatively large enrichment factors), suggesting that high-fat diet exerted its influence on the tumor development of endometrial cancer from the gene molecule transcription level. Therefore, we speculate that high-fat diet activates estrogen signaling pathway, upregulate ER protein expression in endometrial cancer tumor tissues and promote tumor growth of endometrial cancer. This speculation was confirmed by Western blot assay showing that high-fat diet increased ERα protein expression in tumor tissues.

In summary, our group successfully constructed an endometrial cancer PDOX animal model in the nude mouse uterus using subcutaneously amplified tumor tissue blocks, investigated the effects of a high-fat diet on the development of endometrial cancer, provided a reliable method for the in vivo study of the cancer promoting mechanisms of endometrial cancer, helped clinicians give reasonable dietary advice to patients with endometrial cancer, and provided a theoretical basis for the prevention of endometrial cancer in women. Of course, the present paper has certain limitations. In this study, to explore the estrogen pathway and ensure the experimental results are representative, we selected only the tumor tissues with endometrioid adenocarcinoma and ER+ for transplantation. Follow up should expand the sample size of patient-derived endometrial cancer tumor tissues, or be grouped according to the latest molecular typing of endometrial cancer, in order for the experimental results to be more widely representative and convincing. The detailed mechanism regarding the effect of high-fat diet on endometrial cancer still requires further investigation.

## Conclusion

In this study, the PDOX animal model of ER-positive endometrial carcinoma was successfully constructed. The model can grow widely in the uterus and form tumors, and is consistent with human tumors in terms of histomorphology and molecular characteristics. Current research shows that high-fat diet can promote tumor growth, up-regulate genes in estrogen signaling pathway, and increase the expression of ER protein in tumor tissue. This indicates that the model can be used to study the factors affecting the growth of endometrial cancer and the mechanism of promoting cancer in vivo.

### Supplementary Information

Supplementary Figures.

## Data Availability

RNA-seq data have been deposited to National Center for Biotechnology Information Sequence Read Archive (Sequence Read Archive accession: PRJNA915752, ORCID account (0000 0001 9659 4541) and password (mishik987?)). (https://submit.ncbi.nlm.nih.gov/subs/bioproject/SUB12484225/overview).
