# Supplementary material for: High-fat diet promotes tumor growth in the patient-derived orthotopic xenograft (PDOX) mouse model of ER positive endometrial cancer
Source: Sci Rep. 2023 Oct 2;13:16537. doi: 10.1038/s41598-023-43797-1 (PMC10545748; doi:10.1038/s41598-023-43797-1)
Supplement: Supplementary file 1 — Supplementary Figures. [file 41598_2023_43797_MOESM1_ESM.pdf]

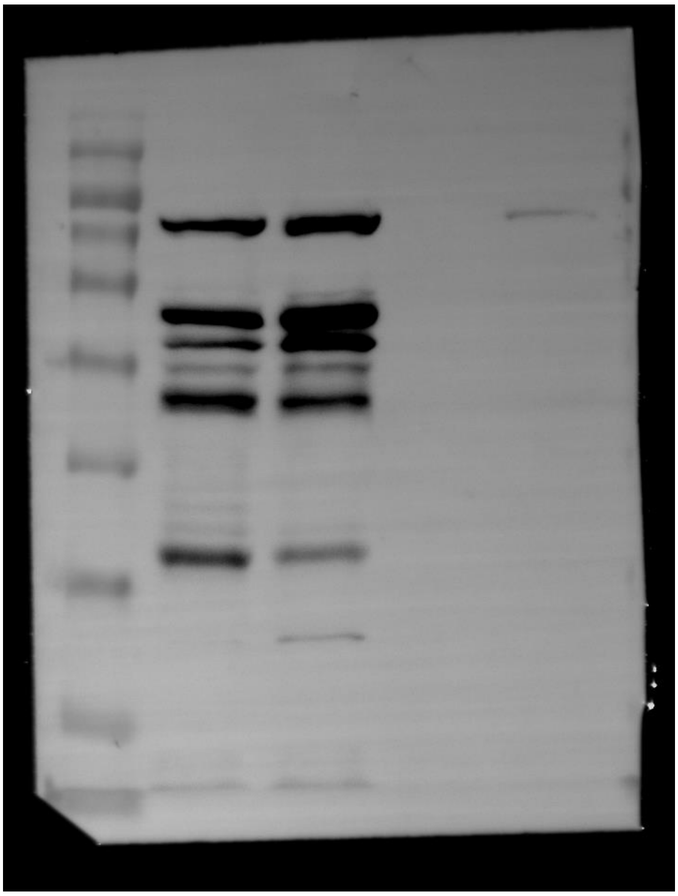

Supplementary Figure S1: the expression of ERα protein in Figure 7

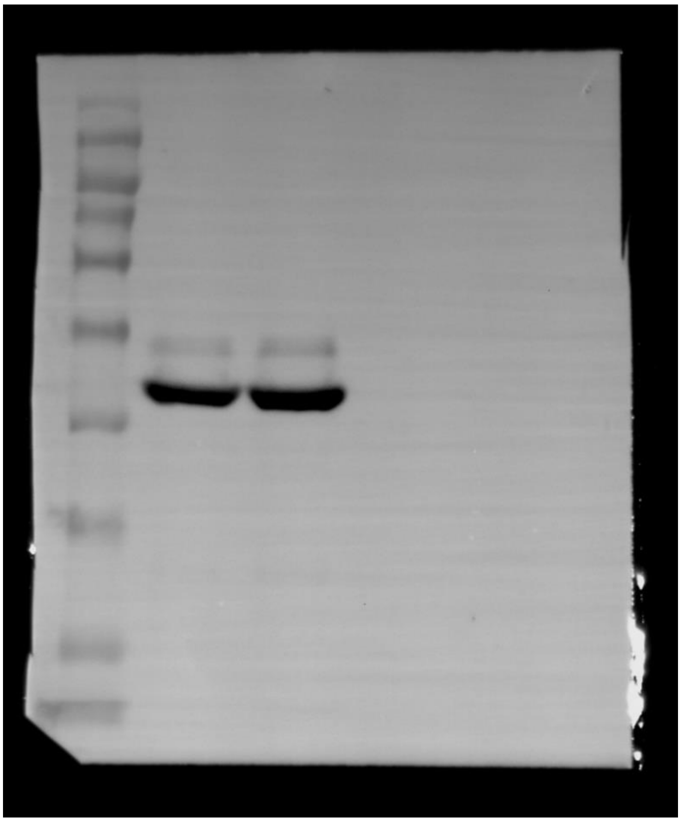

Supplementary Figure S2: the expression of GAPDH protein in Figure 7

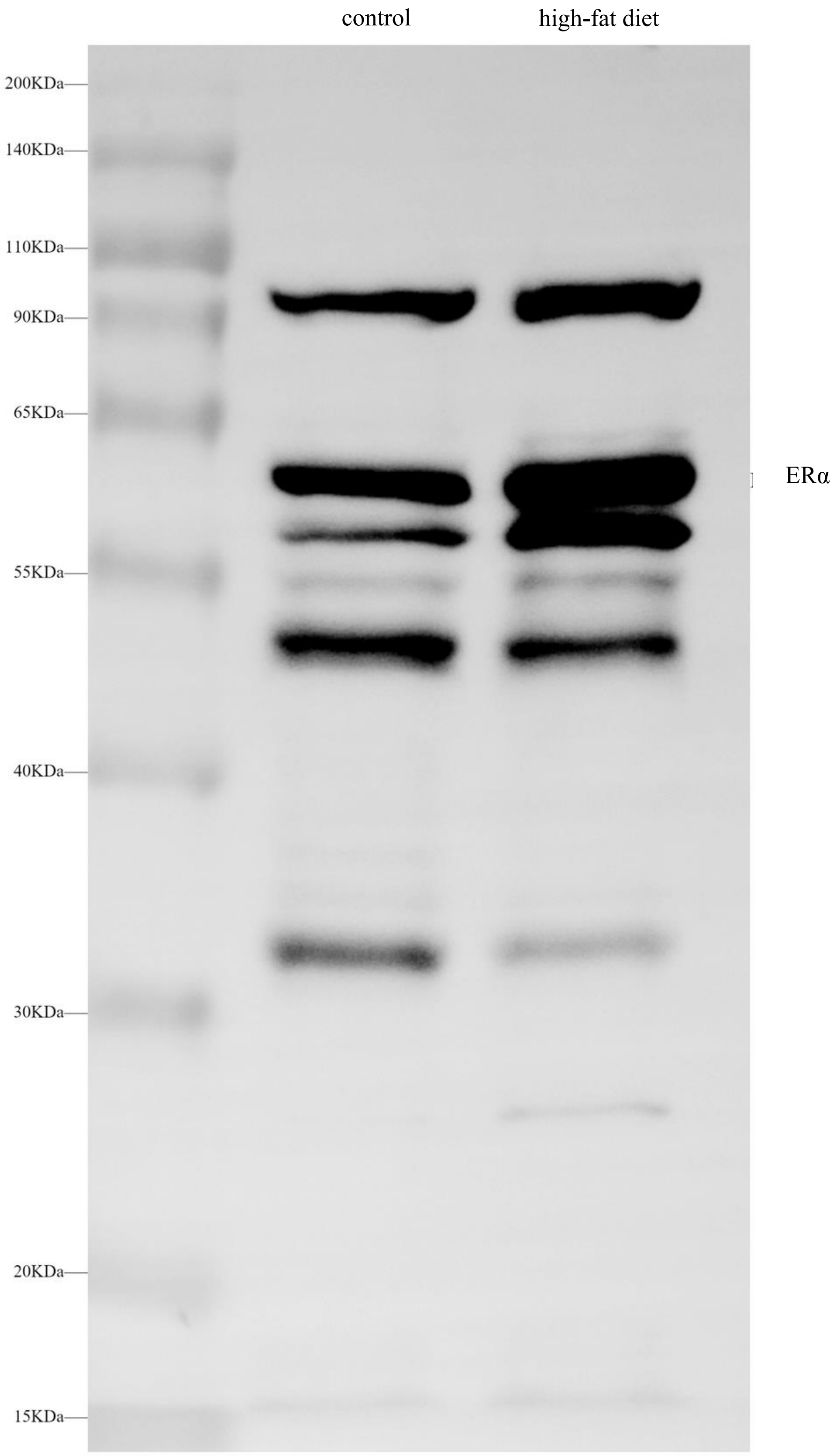

Supplementary Figure S3: the expression of ERα protein in Figure 7 with labels

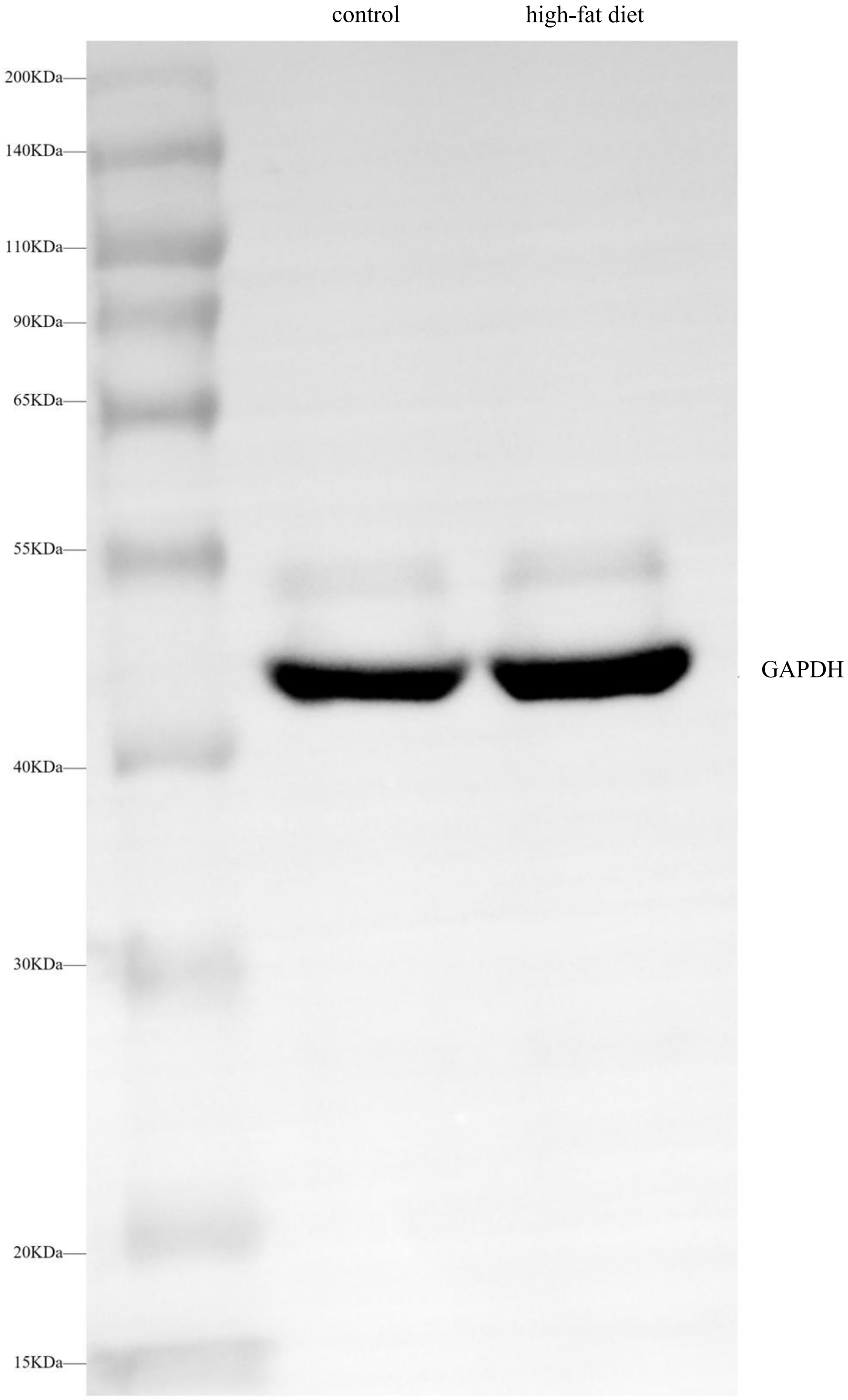

Supplementary Figure S4: the expression of GAPDH protein in Figure 7 with labels

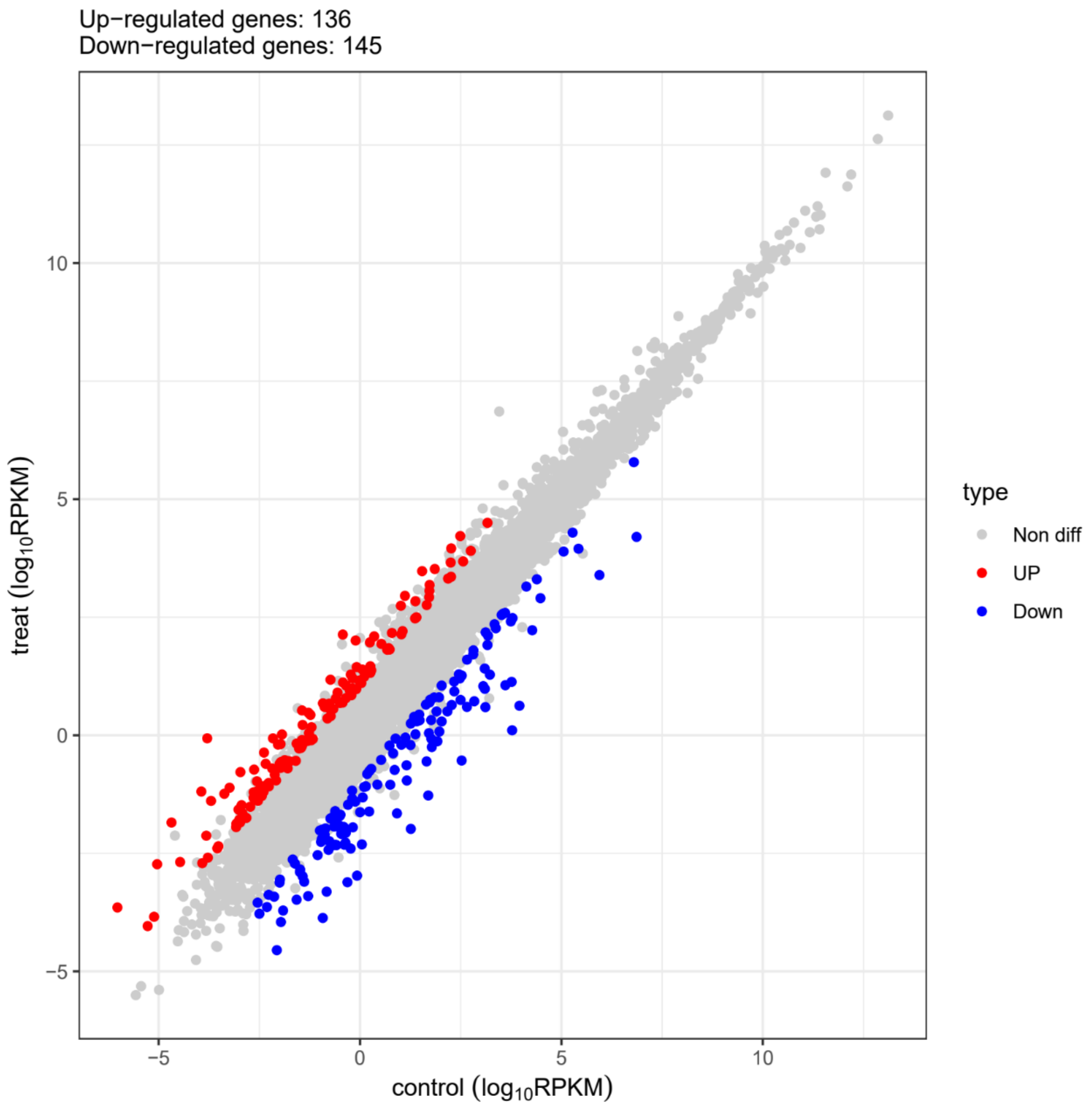

Supplementary Figure S5: Clear image of Figure 6A

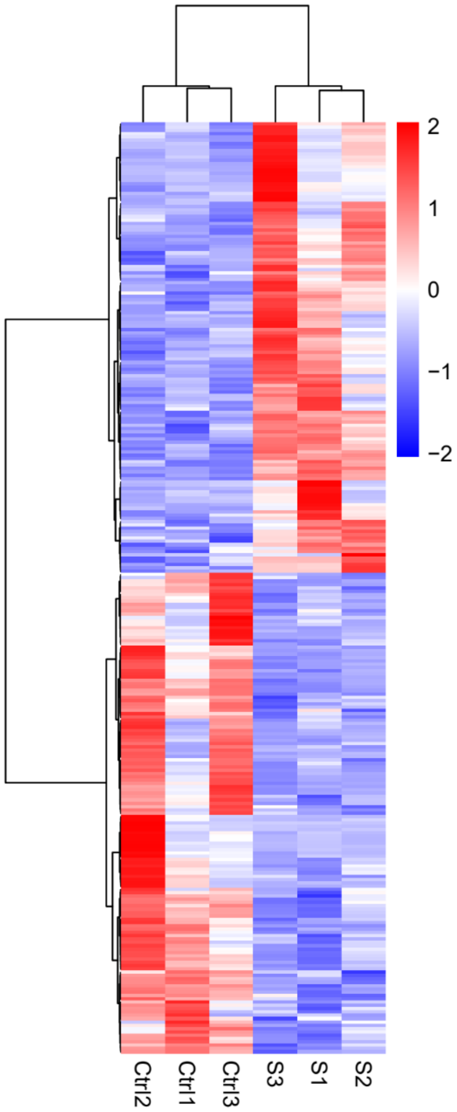

Supplementary Figure S6: Clear image of Figure 6B

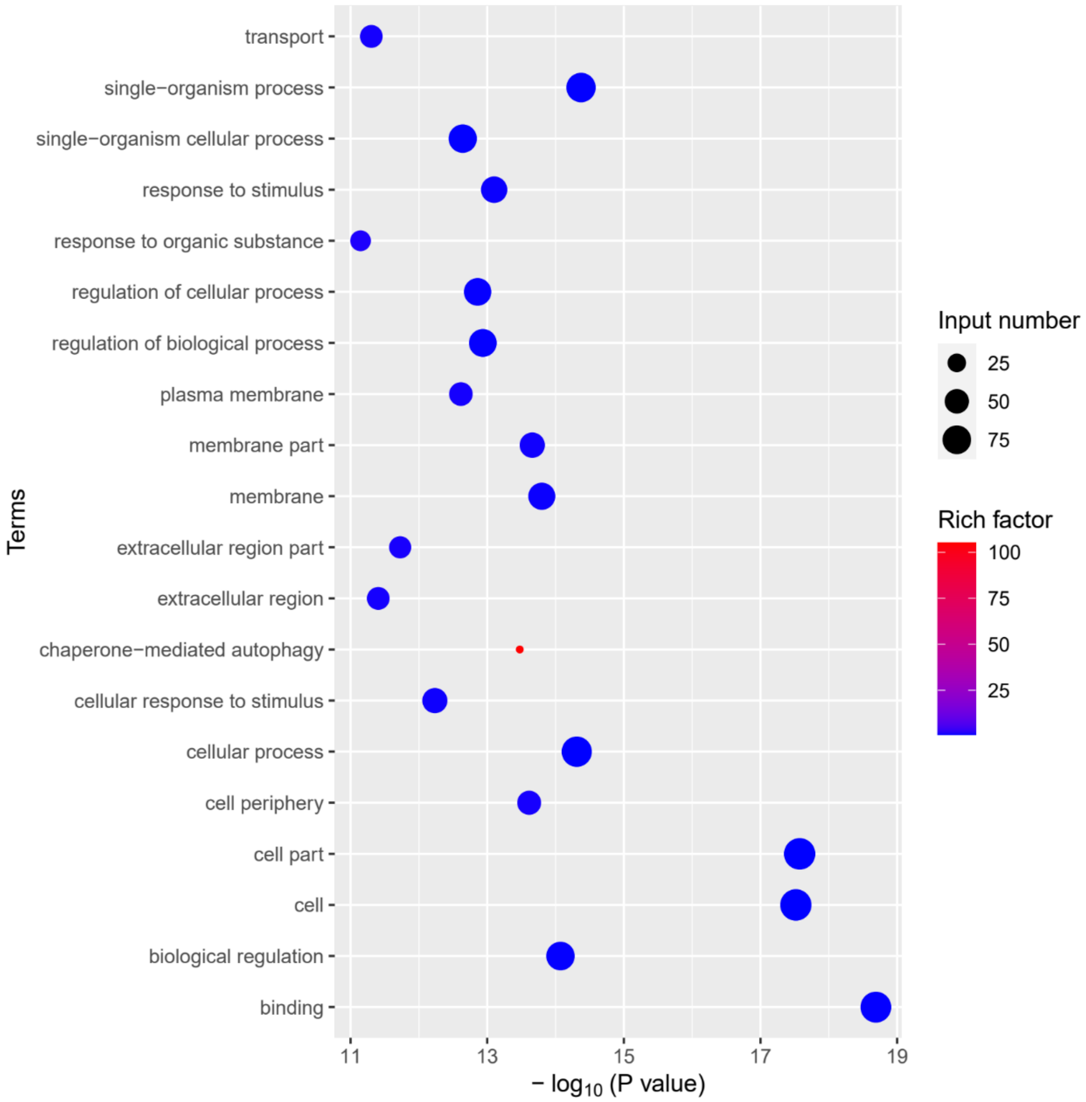

Supplementary Figure S7: Clear image of Figure 6C

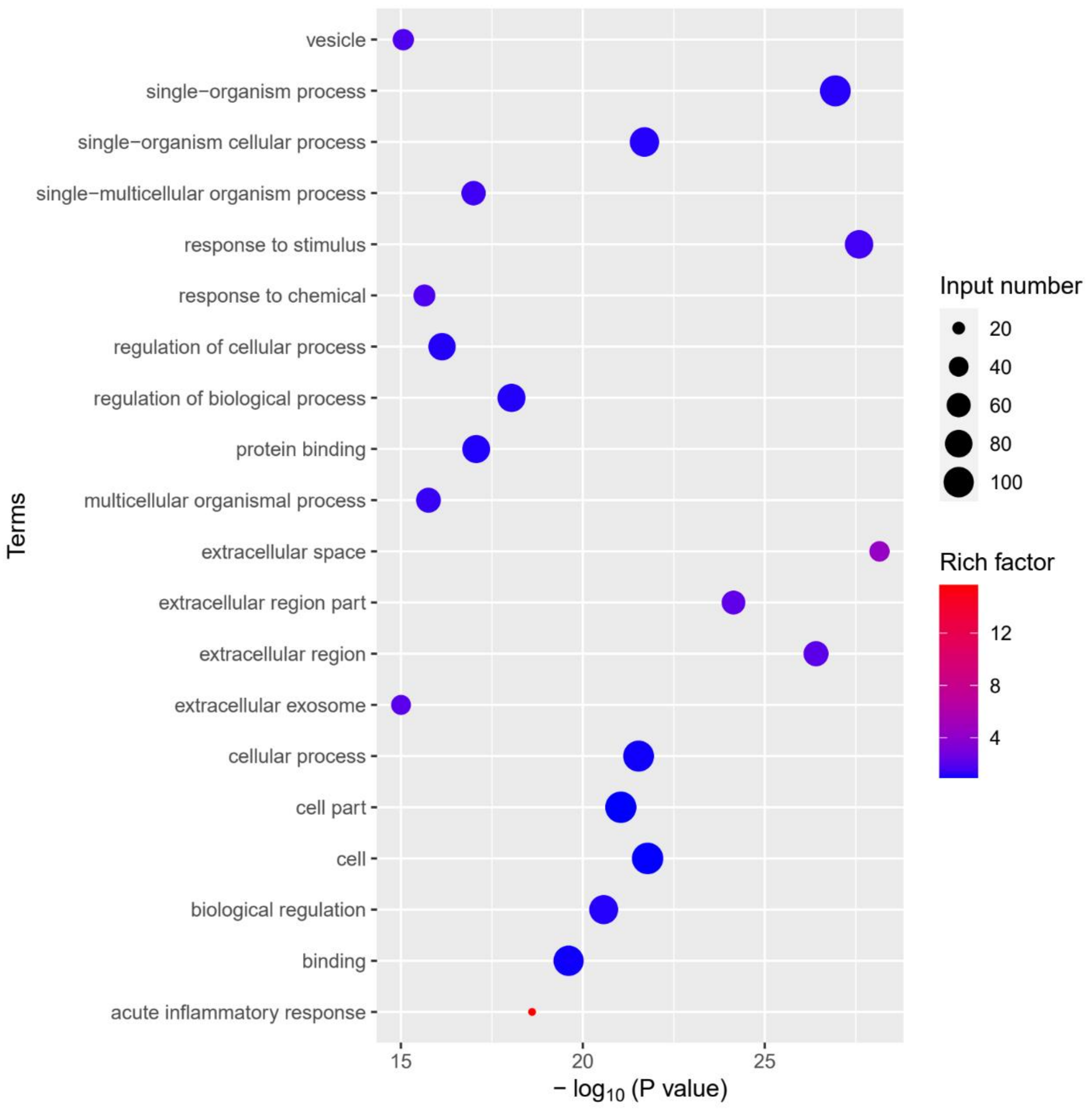

Supplementary Figure S8: Clear image of Figure 6D

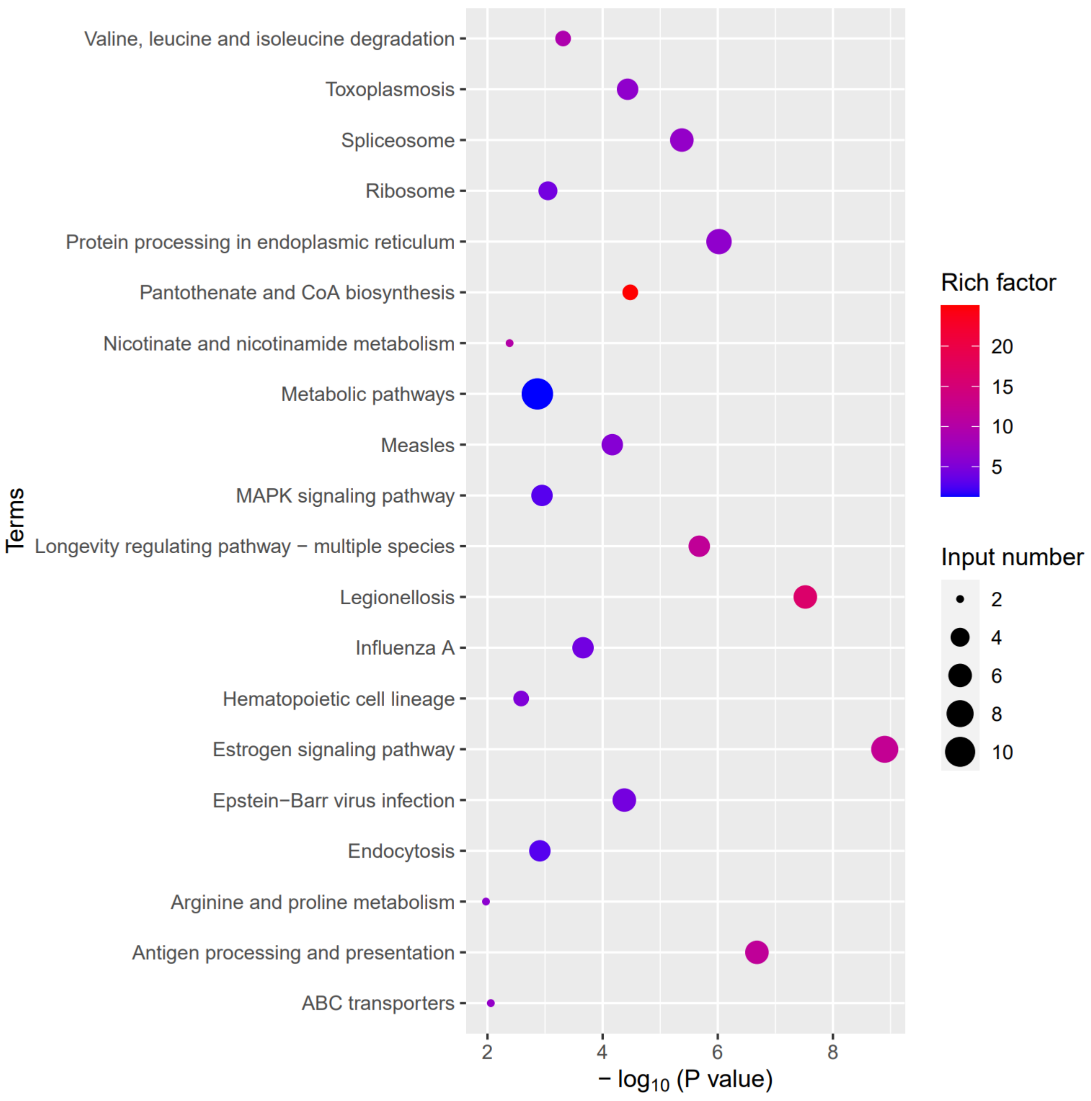

Supplementary Figure S9: Clear image of Figure 6E

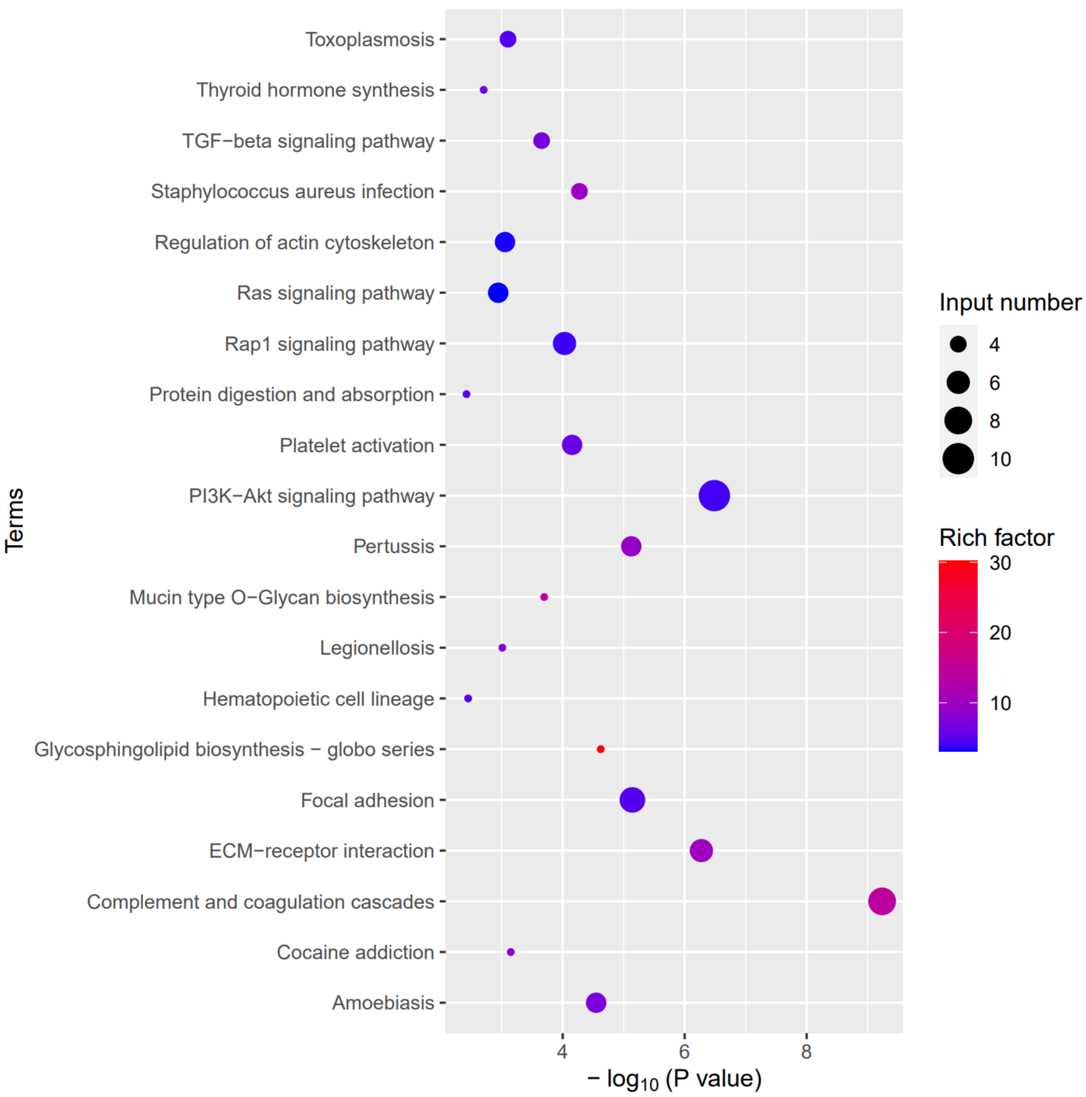

Supplementary Figure S10: Clear image of Figure 6F

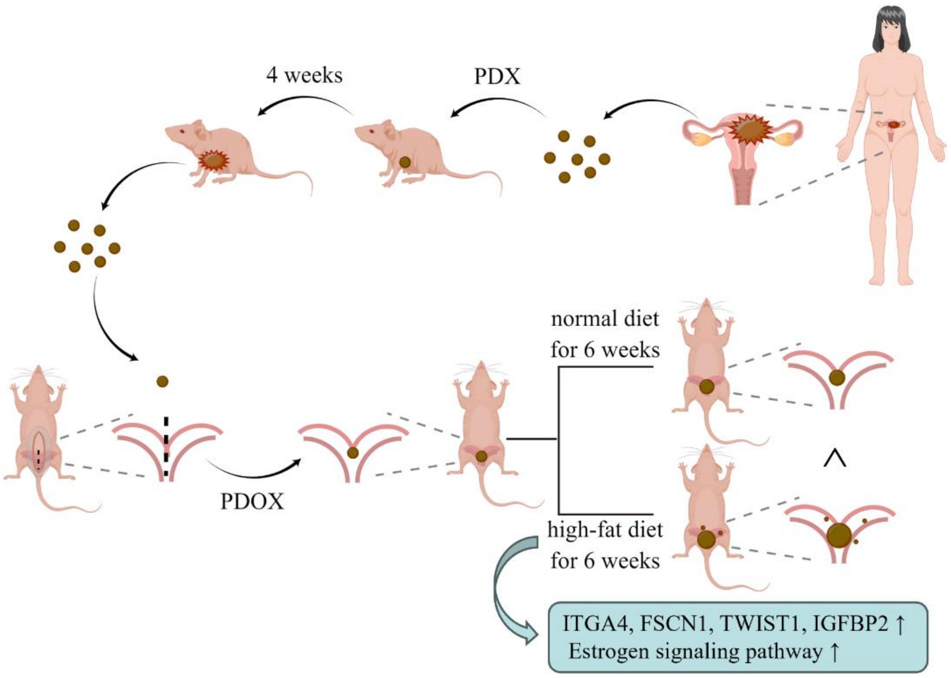

Supplementary Figure S11: Graphical Abstract
